# Testicular and Haematological Cancer Induce Very High Levels of Sperm Oxidative Stress

**DOI:** 10.3390/antiox12061145

**Published:** 2023-05-24

**Authors:** Costanza Calamai, Oumaima Ammar, Viktoria Rosta, Ginevra Farnetani, Salvatore Zimmitti, Lisa Giovannelli, Linda Vignozzi, Csilla Krausz, Monica Muratori

**Affiliations:** 1 Department of Experimental and Clinical Biomedical Sciences “Mario Serio”, University of Florence, 50139 Florence, Italy; costanza.calamai@unifi.it (C.C.); ammaroumayma2014@gmail.com (O.A.); r.viki@hotmail.com (V.R.); ginevra.farnetani@unifi.it (G.F.); linda.vignozzi@unifi.it (L.V.); csilla.krausz@unifi.it (C.K.); 2 Medical Specialization School of Hygiene and Preventive Medicine, University of Florence, 50139 Florence, Italy; salvatore.zimmitti@unifi.it; 3 Department NEUROFARBA, University of Florence, 50139 Florence, Italy; lisa.giovaneli@unifi.it; 4 Andrology, Women’s Endocrinology and Gender Incongruence Unit, AOU Careggi, 50139 Florence, Italy

**Keywords:** sperm oxidative stress, sperm DNA fragmentation, cancer, seminoma, non-seminoma, orchiectomy, flow cytometry

## Abstract

Cancer impairs spermatogenesis, whereas results on sperm DNA integrity are controversial and no data are available about sperm oxidative stress. In cancer patients, we detected sperm DNA fragmentation (sDF) and both viable (ROS production in viable sperm fraction/viable spermatozoa) and total (ROS production in viable sperm fraction/total spermatozoa) oxidative stress. We found that cancer (22.50 (17.00–26.75)%, *n* = 85) increased sDF with respect to the control groups in both normozoospermic subfertile patients (NSP) (12.75 (8.63–14.88)%, *n* = 52, *p* < 0.001) and in healthy donors (HD) (8.50 (7.00–14.00)%, *n* = 19, *p* < 0.001). The induction of viable oxidative stress (*n* = 96) with cancer was even higher: 36.60 (24.05–58.65)% versus 11.10 (8.63–14.90)% in NSP (*p* < 0.001) and 9.60 (8.00–14.03)% in HD (*p* < 0.001). Similar, albeit lower, differences were found for total oxidative stress. SDF sharply correlated to viable oxidative stress when we considered all subjects (cancer patients and controls) (r = 0.591, *p* < 0.001, *n* = 134), but no correlation was found when only cancer patients were studied (r = 0.200; *p* > 0.05, *n* = 63). In conclusion, cancer significantly increases sDF and sperm oxidative stress levels. Additional mechanisms to oxidative attack might be responsible for increased sDF in cancer patients. Because sperm oxidative stress might affect the outcomes of sperm cryopreservation, of cancer treatments and of sperm epigenoma, the detection of oxidative stress could be of help in managing the reproductive issues of cancer patients.

## 1. Introduction

Cancer is a major global disease, and it is anticipated that its incidence will continue to increase in the next two decades for both sexes [1]. For males, subjects younger than 20 and 45 years represent up to 1.1% and 9.2% of cancer patients, respectively [2] and in the range of 15–39 years, the most frequent malignancies are testicular cancer, Hodgkin lymphoma, non-Hodgkin lymphoma and leukaemia [3].

Thanks to the advancement of anticancer therapies and early diagnosis, the survival rate for these types of cancer has become as high as 90% [4], posing the issue of long-term quality of life in survivors. Among the side effects of cancer treatment, the damage to reproductive health is well known, as both chemotherapy and localized radiotherapy can seriously damage spermatogenesis. Indeed, the high cell renewal rate makes germ cells extremely sensitive to these treatments; this highly decreases sperm production and thus provokes temporary or even permanent oligo- or azoospermia [5,6]. In addition to reduced sperm count, several studies have reported post-treatment occurrence of aneuploidies up to two years and increases of sperm DNA fragmentation (sDF) even up to three years from the end of therapies [7,8].

In this scenario, semen cryopreservation prior to undergoing cancer treatment is highly advised in cancer patients [9,10], as frozen samples can be used in assisted reproductive technologies. Although semen cryopreservation itself induces damage to sperm motility, viability and DNA integrity, it currently represents the only option for preserving fertility in males in these instances.

Beyond the effect of gonadotoxic therapies, cancer itself appears to affect spermatogenesis, especially in the case of testicular cancer where common etiological factors are likely involved both in impaired spermatogenesis and cancer development [11]. Van Casteren [12] has reported that only 36% of patients banking semen because of cancer showed normal sperm concentration, with this percentage falling to 28% when patients affected by testicular germ-cell tumours were considered. In addition, 9.7% of such patients were ultimately found to be azoospermic [12]. In a study of 158 men diagnosed with Hodgkin’s lymphoma [13], up to 70% presented with defects in at least one semen parameter, whereas 8% were azoo- or oligoasthenoteratozoospermic. As far as sDF is concerned, studies are more controversial. Indeed, several studies have reported an increase of this type of damage in men affected by testicular and haematological cancer [14,15,16], though others failed to detect such an increase in men with the same types of tumours [17,18]. Controversy also remains when grouping studies according to the technique used to reveal sDF, an aspect that could affect results related to sperm DNA damage [7].

To our best knowledge, no study has investigated the level of oxidative stress in the semen of cancer patients. Oxidative stress is a harmful condition affecting the main functions of sperm and could increase the extent of sperm damage induced by cryopreservation and/or cancer treatment. In addition, attack from reactive oxygen species (ROS) is one of the main mechanisms believed to induce sDF, and thus investigating sperm oxidative stress could lead to insights into the generation of DNA breaks in the spermatozoa of these patients.

The aim of this study was to evaluate whether men affected by testicular and haematological cancer showed higher levels of oxidative stress with respect to normozoospermic subfertile patients and healthy donors. We also analysed sDF and verified whether a relationship occurred between the amount of sperm DNA breakage and the level of oxidative stress.

## 2. Materials and Methods

### 2.1. Reagents and Media

Human Tubal Fluid (HTF) was purchased by Fujifilm, Irvine Scientific (Rome, Italy). Halosperm kit was obtained from Halotech DNA (Madrid, Spain). MitoSOX Red and LIVE/DEAD Fixable Green Dead Cell Stain (LD-G) were obtained from Thermo Fisher Scientific (Waltham, MA USA). All other reagents were obtained from Merck Life Science (Milan, Italy).

### 2.2. Study Population and Semen Collection

Semen samples were collected from cancer patients who were referred to the Semen Cryopreservation and Andrology Laboratory of Careggi Hospital to cryopreserve semen from February 2020 to April 2023. We included subjects who had been affected by testicular (*n* = 79) or haematological cancer (*n* = 44) prior to their undertaking of cryopreservation and their receipt of any session of chemotherapy or radiotherapy. The study was conducted using the remaining semen from the 50 µL aliquot taken to perform routine semen analysis prior to cryopreservation. Subjects with azoospermia and with an insufficient sperm number (<0.05 × 10^6^ million available) were excluded. Oxidative stress and sDF were determined in 96 and 85 of the 123 cancer patients, respectively. In 63 patients it was possible to collect both types of measure. For most patients affected by testicular cancer, information on histological tumour type (*n* = 65) and whether or not they had already undergone unilateral orchiectomy (*n* = 68) was also available.

**Figure 1:**
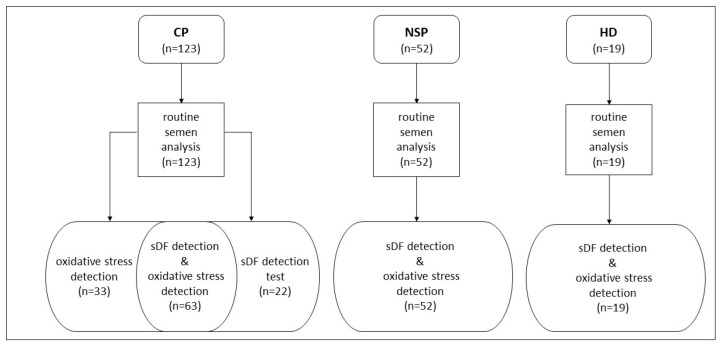
Flowchart showing detection of oxidative stress and sDF in cancer patients and control subjects. CP, cancer patients; NSP, normozoospermic subfertile patients; HD, healthy donors.

For control, we recruited 52 male partners of infertile couples among patients undergoing routine semen analysis and using as criteria of inclusion: (i) normozoospermia and (ii) absence of leukocytospermia, semen viscosity, semen bacteria, smoking habit and recent therapies (from here on indicated as normozoospermic subfertile patients/controls). A second control group was recruited by selecting 19 healthy donors, using as inclusion criteria: (i) absence of any conditions which might induce semen oxidative stress, as assessed by administrating a structured questionnaire: daily sedentary time higher than 8 h/day, professional exposure to toxicants or high temperature, smoking habit, daily alcohol consumption, history of cryptorchidism and varicocele, occurrence of recent (within 6 months) urogenital infections, drug consumption, and current disease; (ii) absence of leukocytospermia, semen viscosity and semen bacteria. Figure 1 reports a flowchart showing detection of oxidative stress and sDF in cancer patients and control subjects.

The study was approved by the ethical committee of AOU Careggi (protocol no. 15693/CAM_BIO) and written informed consent was obtained from participants.

### 2.3. Routine Semen Analysis

Semen analysis was conducted following the WHO guidelines [19] and determined: (i) sperm number and concentration, (ii) sperm progressive and total motility, and (iii) sperm morphology. After proper dilution, sperm concentration was determined in a Neubauer improved cell counting chamber whereas sperm number/ejaculate was obtained multiplying concentration by semen volume. Sperm motility was determined by grading progressive, non-progressive and immotile spermatozoa, in at least 200 cells. For sperm morphology Diff-Quick staining was used and score was done in at least 200 spermatozoa.

### 2.4. Determination of Semen Oxidative Stress

Oxidative stress was determined by MitoSOX Red/LD-G double staining coupled with flow cytometry, as previously reported [20,21]. Briefly, after washing with HTF medium, native semen samples (0.5–2 million of spermatozoa) were incubated with a 1:10,000 dilution of LD-G, for 1 h at RT in the dark in 500 µL of PBS. After washing twice with 200 μL of PBS, samples were split into two aliquots that were incubated (test sample) or not (negative control) with MitoSOX Red 2 µM (15 min at RT in the dark). After double washing with PBS, cells were resuspended in 400 µL of PBS for acquisition with a flow cytometer FACScan (BD Biosciences, San Jose, CA, USA) equipped with a 15-mW argon-ion laser for excitation. After proper fluorescence compensation, LD-G was revealed by an FL-1 detector (515–555 nm wavelength band), whereas MitoSOX Red and propidium iodide (PI, see below) was detected by an FL-2 detector (563–607 nm wavelength band). For each sample, 5000 viable spermatozoa were recorded, gating LD-G negative events within an FSC/SSC flame-shaped region (FR). FR contains spermatozoa and apoptotic bodies and excludes debris and all non-sperm cells [22]. We calculated oxidative stress as the percentage of viable spermatozoa with MitoSOX Red staining on total viable spermatozoa (hereon indicated as viable oxidative stress). For this calculation, after setting quadrants in the MitoSOX Red/LD-G dot plot of negative control including about 99% of events in the LL quadrant (Figure S1A, upper panel), we copied quadrants in the dot plot of the corresponding test sample. Hence, we determined the percentage of the events in the LR quadrant on total events in LL and LR quadrants (Figure S1A, lower panel). We also calculated the percentage of viable spermatozoa with MitoSOX Red staining on total (viable and non-viable) spermatozoa (hereon indicated as total oxidative stress). Total spermatozoa were determined after improved separation of the spermatozoa from semen apoptotic bodies (both contained in FR). To this aim, after flow cytometry acquisition, the negative control (i.e., sample stained with only LD-G) was treated with digitonin (200 mg/mL) and PI (30 mg/mL) and then acquired again by flow cytometer. Figure S1B shows the LD-G/PI dot plot obtained after gating FR region. As shown, PI staining sharply separated apoptotic bodies from spermatozoa (in the grey box, Figure S1B) thus allowing the exact calculation of their amount.

### 2.5. Determination of sDF

sDF was determined with a sperm chromatin dispersion (SCD) test using Halosperm kit and following the manufacturer’s instructions with some modifications. Briefly, after resuspending 50,000 spermatozoa in 1% low melting point agarose, we layered the sample on pre-coated agarose slides. After covering them with a coverslip we placed the slides at 4 °C for a number of minutes. Hence, the slides were treated with an acid denaturation solution and then with a lysing solution, both provided as part of the Halosperm kit. After dehydration with 70% and then 100% ethanol, slides were stained with eosin and then thiazine (15 min at RT for each stain). After drying, slides were examined and sDF scored by counting spermatozoa without or with small halo in a minimum of 200 spermatozoa/sample [23].

### 2.6. Statistical Analyses

Data were analysed by Statistical Package for the Social Sciences for Windows (SPSS 28, Inc., Chicago, IL, USA) and PASS software (PASS 2022, v22.0.2, NCSS, LLC, Kaysville, UT, USA). A Kolmogorov–Smirnov test was used to check the normal distribution of all variables. Since most variables showed a non-normal distribution, data were expressed as median (interquartile range, IQR). To assess whether there were statistically significant differences between cancer patients and the two control groups in age, abstinence, semen parameters, oxidative stress and sDF we used the Kruskal–Wallis test followed, in case of significant difference, by pairwise-adjusted comparisons according to Dunn–Bonferroni. The same statistical tests were used to assess the differences between testicular cancer patients, haematological cancer patients and control groups. The Mann–Whitney U test was used to compare the subtypes of testicular cancer and subjects with and without orchiectomy. Bivariate correlations between the oxidative stress and sDF amounts were evaluated by calculating the Pearson’s correlation coefficient (r), after proper logarithmic transformation of data. The comparisons between (i) cancer patients and normozoospermic subfertile patients and (ii) cancer patients and healthy donors were sized considering viable oxidative stress as the primary endpoint. Preliminary determinations of viable oxidative stress in the three groups of subjects indicated that the unpaired difference was 25.09 ± 20.69% (mean ± SD) (cancer patients vs. normozoospermic subfertile controls) and of 27.40 ± 21.49% (mean ± SD) (cancer patients vs. healthy donors). Hence, to detect the above indicated differences using a Mann–Whitney U test, the number of subjects to be recruited to achieve a power of 0.90 with a significance level of 0.05 was: 17 normozoospermic subfertile patients, 15 healthy donors and 17 cancer patients (the higher number obtained by the two calculations).

## 3. Results

**Figure 2:**
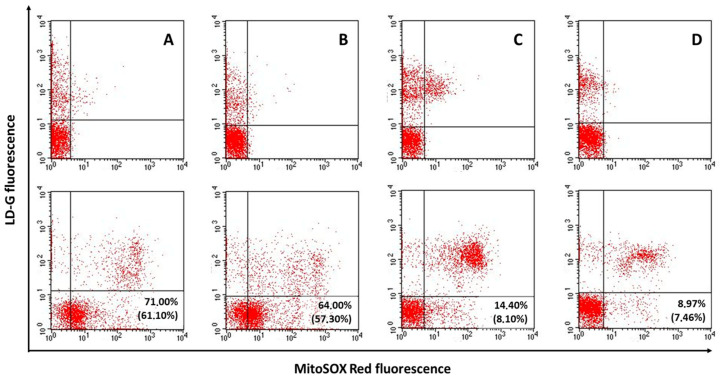
Oxidative stress in semen samples from cancer patients. Representative dot plots of double staining with MitoSOX Red and LD-G in a patient with haematological (**A**) and testicular (**B**) cancer. In (**C**,**D**), dot plots refer to a normozoospermic subfertile patient and a healthy donor, respectively. The percentage of viable oxidative stress is reported for each example (in brackets, the percentage of total oxidative stress). Quadrant setting was established on the corresponding negative controls (first row).

In Table 1, we compared the semen quality of cancer patients with that of control groups. As shown, we found that in cancer patients all main semen parameters were worse with respect to both normozoospermic subfertile patients and, with the exception of sperm morphology, healthy donors. Cancer patients were younger than normozoospermic subfertile patients but with similar age of healthy donors (Table 1). These results were confirmed when we separately compared testicular and haematological cancer patients to control groups (Table S1), however haematological subjects showed similar values as healthy donors for sperm concentration, number and normal morphology. In addition, no significant difference in age was observed between these patients and both control groups (Table S1). No significant difference in conventional semen parameters and age was found between the two types of cancer (Table S1). In Table S2, we report the frequency in cancer patients of the following characteristics which could affect both semen oxidative stress and sDF: leukocytospermia, semen bacteria and viscosity, smoking habit, and recent therapies. None of these conditions were present in control groups because of the recruitment criteria (see also below). To detect semen oxidative stress, we used a double staining with MitoSOX Red and LD-G [20,21] coupled with flow cytometry. This technique allowed us to reveal oxidative stress in viable spermatozoa of native semen samples and express the parameter as percentage on viable (viable oxidative stress) and total (total oxidative stress) spermatozoa (see M&M for details). Figure 2 reports typical MitoSOX Red/LD-G dot plots as obtained in a patient with haematological (Figure 2A) and testicular (Figure 2B) cancer and in a normozoospermic subfertile control (Figure 2C) and a healthy donor (Figure 2D).

**Figure 3:**
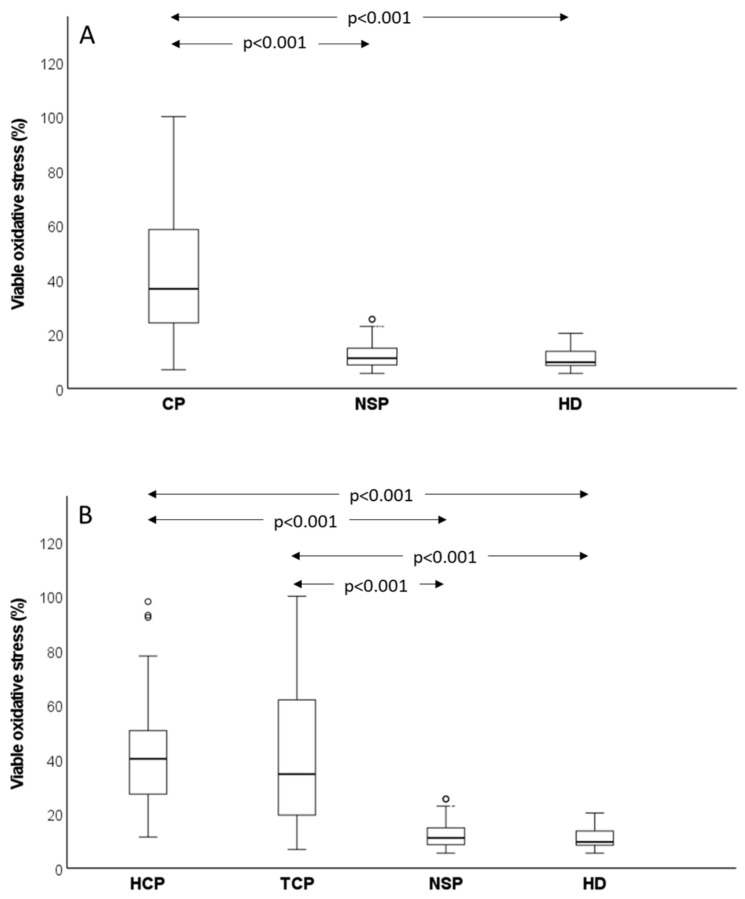
Box graphs reporting median values (IQR) of viable oxidative stress. Comparison between control groups (NSP, normozoospermic subfertile patients and HD, healthy donors) and all cancer patients (**A**) or testicular and haematological cancer patients (**B**). Pairwise-adjusted comparisons according to Dunn–Bonferroni. Circle = mild outlier.

**Figure 4:**
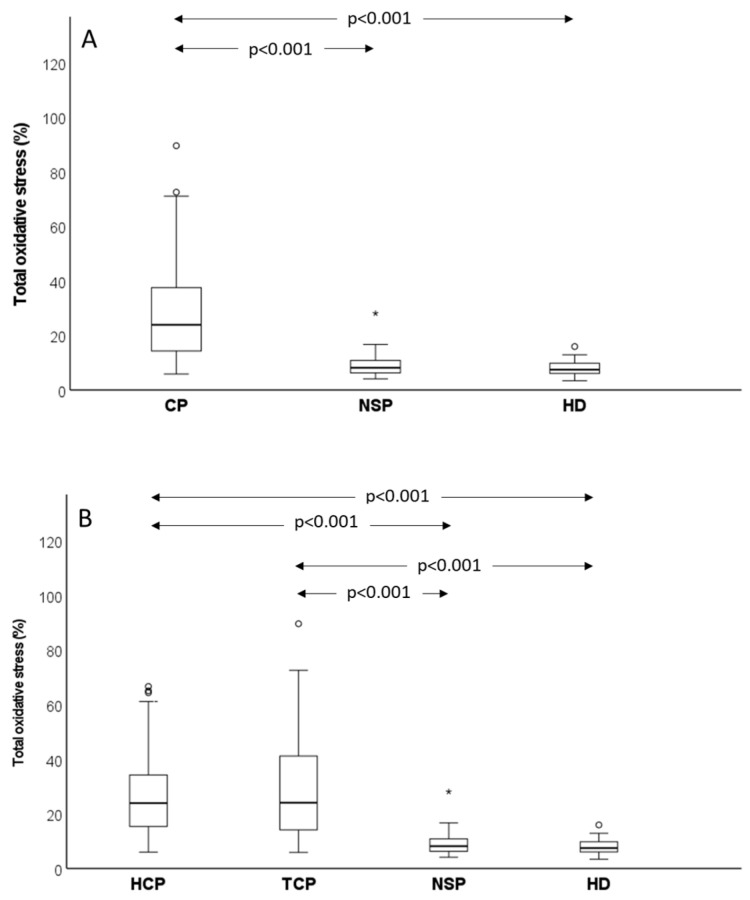
Box graphs reporting median values (IQR) of total oxidative stress. Comparison between control groups (NSP, normozoospermic subfertile patients and HD, healthy donors) and all cancer patients (**A**) or testicular and haematological cancer patients (**B**). Pairwise-adjusted comparisons according to Dunn–Bonferroni. Circle = mild outlier; asterisk = extreme outlier.

As shown, cancer patients exhibited dramatically higher values of viable and total oxidative stress than control subjects, and this result was confirmed by comparing median values observed in cancer patients (*n* = 96) and control groups (normozoospermic subfertile patients, *n* = 52, *p* < 0.001; healthy donors, *n* = 19, *p* < 0.001) (Figure 3A). Similar results were obtained when total oxidative stress was evaluated, albeit the difference between cancer patients and controls (*p* < 0.001 vs. both normozoospermic subfertile patients and healthy donors) was slightly lower for this parameter (Figure 4A). When testicular and haematological cancer were compared separately to control groups, similar results were found for viable (Figure 3B) and total (Figure 4B) oxidative stress in the two types of cancer. We did not observe any difference either in viable or in total oxidative stress nor between normozoospermic subfertile patients and healthy donors (Figure 3 and Figure 4), though slightly higher values of viable oxidative stress were observed in the former (respectively: 11.10 (8.63–14.90)% vs. 9.60 (8.00–14.03)%, *p* > 0.05) (Figure 3).

**Figure 5:**
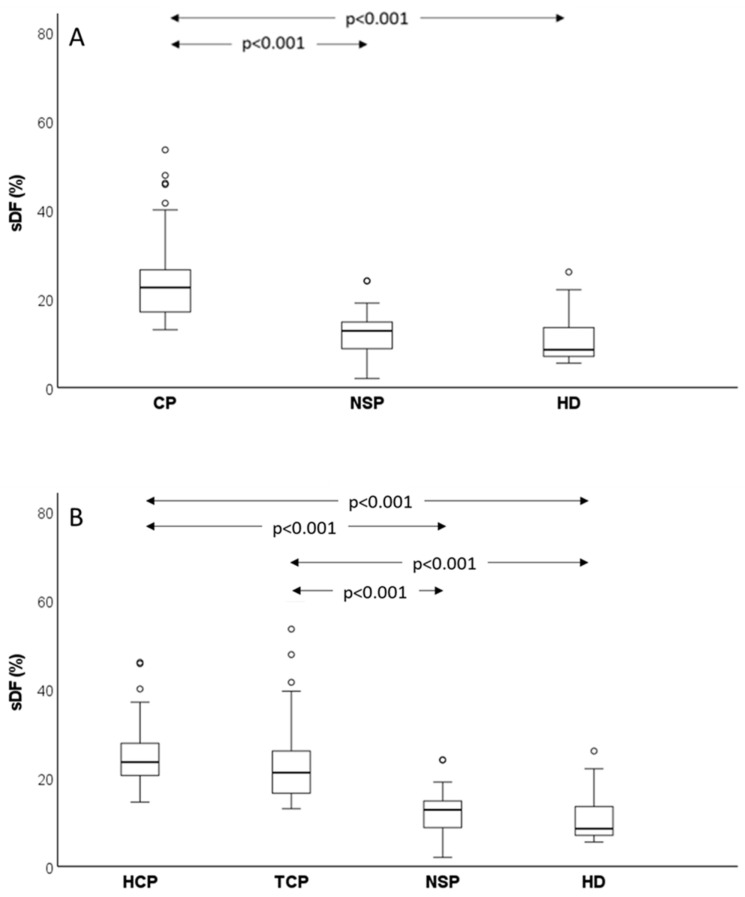
Box graphs reporting median values (IQR) of sDF. Comparison between control groups (NSP, normozoospermic subfertile patients and HD, healthy donors) and all cancer patients (**A**) or testicular and haematological cancer patients (**B**) Pairwise-adjusted comparisons according to Dunn–Bonferroni. Circle = mild outlier.

SDF was measured by SCD test in 85 out of 123 cancer patients and in control subjects and the obtained results are consistent with oxidative stress determination. Indeed, the amount of sperm DNA damage was much higher in cancer patients than in normozoospermic subfertile controls (*p* < 0.001) and healthy donors (*p* < 0.001) (Figure 5A). Further, when we separately considered testicular and haematological cancer, both showed increased sDF with respect to controls (Figure 5B). No difference was observed either between the two types of cancer or between the two control groups, though a trend towards higher values of sDF was observed in normozoospermic subfertile patients (12.75 (8.63–14.88)%) with respect to healthy donors (8.50 (7.00–14.00)%, *p* > 0.05) (Figure 5).

As mentioned above, several conditions were present in cancer patients and not in control groups, which might affect the level of both semen oxidative stress and sDF (Table S2). To verify whether the increases of oxidative stress and sDF in cancer were affected by these conditions, we excluded subjects with at least one of these characteristics: leukocytospermia, semen bacteria, semen viscosity, smoking habit and recent therapies. Results indicate that the remaining 33 cancer patients still showed much higher values of total (32.03 (19.00–54.59)%, *n* = 23, *p* < 0.001) and viable (46.41 (25.60–71.60)%, *n* = 23, *p* < 0.001) oxidative stress and sDF (23.00 (17.00–26.00)%, *n* = 27, *p* < 0.001) than controls (respectively: 8.00 (6.13–10.40)%; 10.70 (8.60–14.60)%; and 12.00 (8.00–14.50)%, as calculated in total control subjects, *n* = 71).

**Figure 6:**
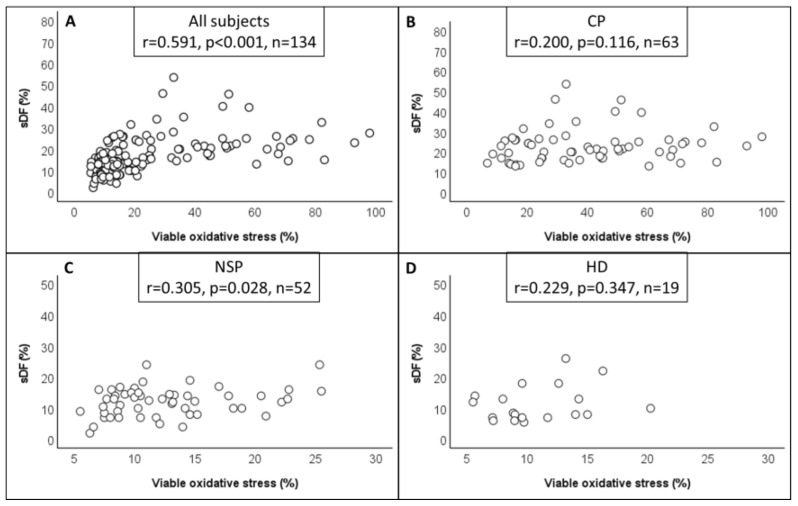
Relationship between viable oxidative stress and sDF. Dispersion plots reporting sDF against viable oxidative stress in total subjects (**A**), cancer patients (CP) (**B**), normozoospermic subfertile patients (NSP) (**C**) and healthy donors (HD) (**D**). In each panel, Pearson’s correlation coefficient (r) is shown.

To verify whether there was an association between the levels of oxidative stress and sDF, we performed a correlation analysis between the two parameters. When we considered all subjects (*n* = 134) we found a sharp correlation between sDF and both viable (r = 0.591, *p* < 0.001) (Figure 6A) and total oxidative stress (r = 0.486, *p* < 0.001) (data not shown). The amounts of sDF also correlated with viable oxidative stress when we considered normozoospermic subfertile patients (r = 0.305, *p* < 0.05, *n* = 52) separately (Figure 6B), while the correlation with total oxidative stress was not statistically significant (r = 0.194, *p* > 0.05, *n* = 52) (data not shown). Surprisingly, when only cancer patients were considered (*n* = 63), sDF did not correlate with either viable (r = 0.200; *p* > 0.05) (Figure 6C) or total oxidative stress (r = −0.066, *p* > 0.05) (data not shown). No correlation was found between sDF and either viable oxidative stress (r = 0.229, *p* > 0.05) (Figure 6D) or total oxidative stress (r = 0.069, *p* > 0.05) (data not shown) when healthy donors (*n* = 19) were considered separately, possibly because of small sample size.

Information on histology and surgery before treatment was available for most testicular patients. When we compared two main subtypes of testicular cancer, we found a higher age (*p* < 0.001) in seminoma (35.00 (29.00–37.50, *n* = 33)) vs. non seminoma (25.00 (20.00–30.00, *n* = 23)) but no difference in conventional semen parameters, SCD and oxidative stress (data not shown). Similarly, no difference was found when we compared subjects with (*n* = 29) and without (*n* = 39) orchiectomy (data not shown), although surgery tended to decrease sperm count (million/ejaculate) (with orchiectomy: 56.16 (19.75–148.10) vs. without orchiectomy: 118.00 (40.92–255.60), *p* = 0.050), sperm concentration (million/mL) (with orchiectomy: 25.00 (5.55–61.25) vs. without orchiectomy: 40.50 (24.00–78.00), *p* = 0.083) and sperm normal morphology (with orchiectomy: 4.00 (2.00–6.00)% vs. without orchiectomy: 6.00 (3.00–7.00)%, *p* = 0.093). We then studied the effect of orchiectomy, after separating seminoma from non-seminoma (Table S3). In seminoma, we found a sharp, albeit not significant (*p* = 0.120), decrease of viable oxidative stress following surgery (with orchiectomy: 20.47 (14.00–45.90)%, *n* = 11 vs. without orchiectomy: 39.05 (26.77–68.72)%, *n* = 14)). Similar results were found also for non-seminoma, when we compared subjects with orchiectomy (36.89 (16.28–60.50)%, *n* = 7) to those without orchiectomy (63.17 (25.80–69.35)%, *n* = 9) (*p* = 0.351). A trend toward the reduction of sperm concentration and count after surgery was also observed, as expected (Table S3). This analysis also showed that grouping subjects according to the presence/absence of orchiectomy, non-seminoma had much higher, albeit not significant (in subjects with orchiectomy, *p* = 0.781; in subjects without orchiectomy, *p* = 0.375), levels of viable oxidative stress than seminoma (Table S3).

## 4. Discussion

In this study we observed increased levels of sperm oxidative stress, beyond poorer semen quality and increased sDF, in patients with haematological and testicular cancer. In cancer patients we failed to detect a significant correlation between oxidative stress and sDF, suggesting that other mechanisms, beside ROS attack, might be involved in inducing sperm DNA damage in these subjects. Future studies are needed to clarify whether detection of sperm oxidative stress will be of help in managing the consequences of cancer on male fertility.

Many previous studies have reported that cancer itself has a negative impact on semen quality [24,25], though some investigations have found that the impairment of spermatogenesis depends on the cancer type [25,26]. In this study we found poorer semen parameters in both haematological and testicular cancer than in control subjects and it is notable that subjects with azoospermia or in which the available sperm number was insufficient (<0.05 × 10^6^ million) were excluded from the study. We also found that cancer patients, prior to receiving any session of chemotherapy or radiotherapy, showed increased levels of sDF, in agreement with many previous studies [14,15,16] but not confirmed by others [17,18]. Controversy also remains after grouping studies according to the technique used to reveal sDF [7], suggesting that differences in the recruitment criteria and in sample size are likely to be responsible for the contradictory results. To our best knowledge, this is the first study reporting that sperm oxidative stress is also increased in patients with both haematological and testicular cancer, though a large variability was observed among the patients.

It is not clear how cancer impairs spermatogenesis, but local and systemic mechanisms have been proposed which could play different roles depending on the subject [27]. For testicular cancer, pre-existing defects could lead to both cancer and poor semen quality, beyond cryptorchidism and hypospadias, according to the proposed testicular dysgenesis syndrome [11]. In addition, secretion of factors, including hormones, by cancer cells could affect spermatogenesis via a paracrine way [28] or by altering the hypothalamo–pituitary–gonadal axis and thus the normal hormonal milieu of seminiferous epithelium [6]. A hormonal systemic unbalance due to stress response, direct infiltration of the central nervous system and endocrinopathies have also been seen to be involved in sperm dysfunction in cases of haematological cancer [29]. In addition, according to several reports [30,31,32,33], other systemic symptoms of cancer, including fever, could also play a role in disturbing spermatogenesis.

Some of the proposed mechanisms to explain the impairment of spermatogenesis can also account for the increase of sDF observed with cancer. Besides the well-known relation between increased levels of sDF and poor spermatogenesis [34], it has been reported that sperm DNA breakage may be associated with a history of cryptorchidism [35], fever [36] and disturbed testis hormone levels [37,38]. In addition, a role has been recently proposed [30] for the unbalanced testicular cytokines and growth factors produced by testis interstitial tissue [39], cancer cells themselves [40,41] and by resident [42] or infiltrating [43,44] immune system cells. Among these molecules, many are known for their involvement not only in proliferation and differentiation of spermatogonial stem cells but also in testis apoptosis [40], one of the main processes responsible for sDF [45].

As mentioned, cancer patients exhibit very high levels of sperm oxidative stress. Given the well-known association between inflammation and oxidative stress [46], a local and/or systemic perturbation of cytokines increasing the pro-inflammatory cytokines, could explain this finding. In agreement with this hypothesis, an overexpression of IL-1β, IL-6 and TNF-α [40,47,48] has been reported in seminoma. Increased amounts of cytokines [49], including the pro-inflammatory IL-6 and IL-8 [41], altogether with the frequent infiltration of cancer cells in testis [30], has also been observed in patients affected by leukaemia. Similarly, stemming from the finding that inflammatory symptoms affect semen quality, an involvement of pro-inflammatory cytokines has also been proposed for lymphoma [13,50]. Since the role of cytokines in the pathophysiology of the male reproductive tract is under intense investigation, future findings will, hopefully, be able to better clarify the link between the perturbation of these inflammatory peptides by cancer and increased oxidative stress.

When we compared seminoma to non-seminoma, we did not find significant differences in semen quality, sDF or oxidative stress levels. Similarly, orchiectomy did not show an effect on the studied parameters. However, when we separately considered seminoma and non-seminoma, subjects who had not yet undergone orchiectomy showed much higher values of viable oxidative stress than those who already had. This difference did was not ultimately statistically significant, possibly due to the small number of subjects, and further studies are needed to address this point which could be of importance to understand which is the best time (before or after surgery) to cryopreserve semen in these subjects (see below for further discussion on this point). When we separately considered subjects with and without orchiectomy, we also found that non-seminoma showed higher levels of viable oxidative stress than seminoma, a finding somehow consistent with the more aggressive characteristics of non-seminoma with respect to seminoma [51]. However, the difference was not significant and has to be confirmed by studying a higher number of subjects.

In this study, oxidative stress was expressed as a percentage of viable spermatozoa showing MitoSOX Red staining, calculated on either viable or total sperm population. Interestingly, the increase of sperm ROS production in cancer patients vs. controls was sharper when viable oxidative stress was considered. This result may be due to the fact that total, but not viable, oxidative stress is decreased by the amount of non-viable spermatozoa, which likely occur at a higher extent in patients with cancer than in controls. Hence, viable oxidative stress was shown to be a more sensitive parameter than total oxidative stress, when comparing cancer patients to controls.

Besides the determination of sDF and sperm oxidative stress, we also verified the relationship between these two sperm traits, finding a sharp correlation when we considered all subjects (cancer patients and controls). Surprisingly, no correlation was observed when we considered only cancer patients. We tend to exclude technical reasons for this result, as we previously found a strict relationship between oxidative stress and sDF as revealed by the two techniques used in this study [20]. In addition, in normozoospermic subfertile patients, sDF did show a clear, albeit weak, correlation with sperm oxidative stress. On the other hand, it is well known that other mechanisms, beside oxidative stress, can be responsible for sperm DNA breakage in native semen samples, including testis apoptosis and/or impairment of chromatin maturation [45], both possibly enhanced in cancer patients. Since this study did not investigate markers of apoptosis and/or chromatin immaturity, further data are needed to address this point.

The huge but variable amounts of oxidative stress found in cancer patients in this study might have important wider implications. First, it is possible that high sperm ROS production negatively affects the outcome of cryopreservation, in terms of the recovery of sperm functions in thawed samples, as has already been reported in a study regarding poor basal semen quality [52]. Oxidative insult is believed to be one of the main mechanisms responsible for decreased motility, viability and DNA integrity during freezing/thawing [53]. In addition, it has already been reported that cancer patients show higher levels of sDF in post-thawing samples than subjects cryopreserving semen for other reasons [54], suggesting that high levels of oxidative stress in basal samples might exacerbate the damage due to sperm freezing/thawing processes. Secondly, it is well known that the consequences of cancer treatment cannot be predicted as they depend on several variables, including individual ones. However, it has been extensively reported that higher levels of endogenous antioxidants could offer a defence against damage caused by gonadotoxic treatments [55]. Conversely, priory injury by oxidative stress might worsen the deleterious effects of cancer therapies. Third, a role for oxidative stress in negatively affecting the sperm epigenome has been proposed [56] and it has recently been reported that cancer may induce pre-treatment aberrant epigenetic marks in spermatozoa [57]. Thus, we can speculate that high levels of sperm oxidative stress are one of the links between cancer and sperm epigenetic modifications. Future studies are needed to explore each of these three novel hypotheses in order to verify whether detection of oxidative stress in cancer patients could be of help in predicting the outcomes of cryopreservation and/or gonadotoxic treatment or even sperm epigenome modifications by cancer.

## 5. Conclusions

In conclusion, we have shown here that both testicular and haematological cancer induce very high levels of sperm oxidative stress. Although both oxidative stress and sDF increased, the lack of a significant correlation between these two parameters suggests that other mechanisms, different from ROS attack, contribute to the induction of sperm DNA breakage in cancer patients. Given that sperm oxidative stress might affect the outcomes of cryopreservation and of cancer treatment and impact on the sperm epigenome, detection of sperm oxidative stress can be a potential biomarker for the more appropriate/personalized management of oncological patients.

## Figures and Tables

**Table 1:** Age, abstinence and main semen parameters in cancer patients, normozoospermic subfertile patients and healthy donors.

Parameter	CP *n* = 123	NSP *n* = 52	HD *n* = 19	*p*-Values	
**Age** **(y)**	33.00 (27.00–37.00)	35.00 (32.25–39.75)	32.00 (26.00–36.00)	0.019 ^1^	0.021 ^2^ 1.00 ^3^ 0.184 ^4^
**Abstinence** **(d)**	4.00 (3.00–6.00)	4.00 (3.00–5.38)	3.00 (2.00–4.00)	0.015 ^1^	1.00 ^2^ 0.033 ^3^ 0.012 ^4^
**Volume** **(mL)**	3.00 (2.10–4.20)	3.65 (2.60–4.48)	3.50 (2.20–4.60)	0.080 ^1^	
**pH**	7.60 (7.40–7.80)	7.60 (7.40–7.75)	7.20 (7.20–7.40)	<0.001 ^1^	0.765 ^2^ <0.001 ^3^ <0.001 ^4^
**Concentration** **(106/mL)**	39.50 (12.00–74.30)	112.50 (72.00–159.50)	94.00 (47.00–102.00)	<0.001 ^1^	<0.001 ^2^ 0.006 ^3^ 0.693 ^4^
**Number** **(106/ejaculate)**	97.20 (40.04–224.20)	395.80 (252.20–620.88)	226.78 (138.56–398.25)	<0.001 ^1^	<0.001 ^2^ 0.004 ^3^ 0.383 ^4^
**Progressive Motility** **(%)**	42.00 (28.00–52.00)	53.00 (46.00–61.75)	56.00 (50.00–64.00)	<0.001 ^1^	<0.001 ^2^ <0.001 ^3^ 1.00 ^4^
**Immotile** **(%)**	46.00 (38.00–58.00)	38.00 (31.50–44.00)	34.00 (30.00–40.00)	<0.001 ^1^	<0.001 ^2^ <0.001 ^3^ 0.784 ^4^
**Normal** **Morphology** **(%)**	5.00 (3.00–7.00)	6.00 (4.25–9.00)	4.00 (4.00–3.00)	<0.001 ^1^	0.004 ^2^ 0.395 ^3^ 0.002 ^4^

CP, cancer patients; NSP, normozoospermic subfertile patients; HD, healthy donors. ^1^ = Kruskal–Wallis test; ^2^, ^3^ and ^4^ = pairwise-adjusted comparisons according to Dunn–Bonferroni for CP vs. NSP, CP vs. HD and NSP vs. HD, respectively. Data are median (IQR).

## Data Availability

The datasets generated during and/or analysed during the current study are available from the corresponding author on reasonable request.

## Supplementary Materials

The following supporting information can be downloaded at: https://www.mdpi.com/article/10.3390/antiox12061145/s1, Figure S1: Flow cytometric detection of viable and total sperm oxidative stress; Table S1: Age, abstinence and main semen parameters in testicular and haematological cancer patients and in normozoospermic subfertile patients and healthy donors, Table S2: Occurrence of leukocytospermia, semen bacteria and viscosity, smoking habit and recent therapies in cancer patients; Table S3: Age, abstinence, conventional semen parameters, viable and total oxidative stress and sDF in subjects affected by seminoma and non-seminoma with and without orchiectomy.
