# Supplementary material for: Testicular and Haematological Cancer Induce Very High Levels of Sperm Oxidative Stress
Source: Antioxidants (Basel). 2023 May 24;12(6):1145. doi: 10.3390/antiox12061145 (PMC10294921; doi:10.3390/antiox12061145)
Supplement: Supplementary file 1 [file antioxidants-12-01145-s001.zip › antioxidants-2398458-supplementary.pptx]

## Slide 1
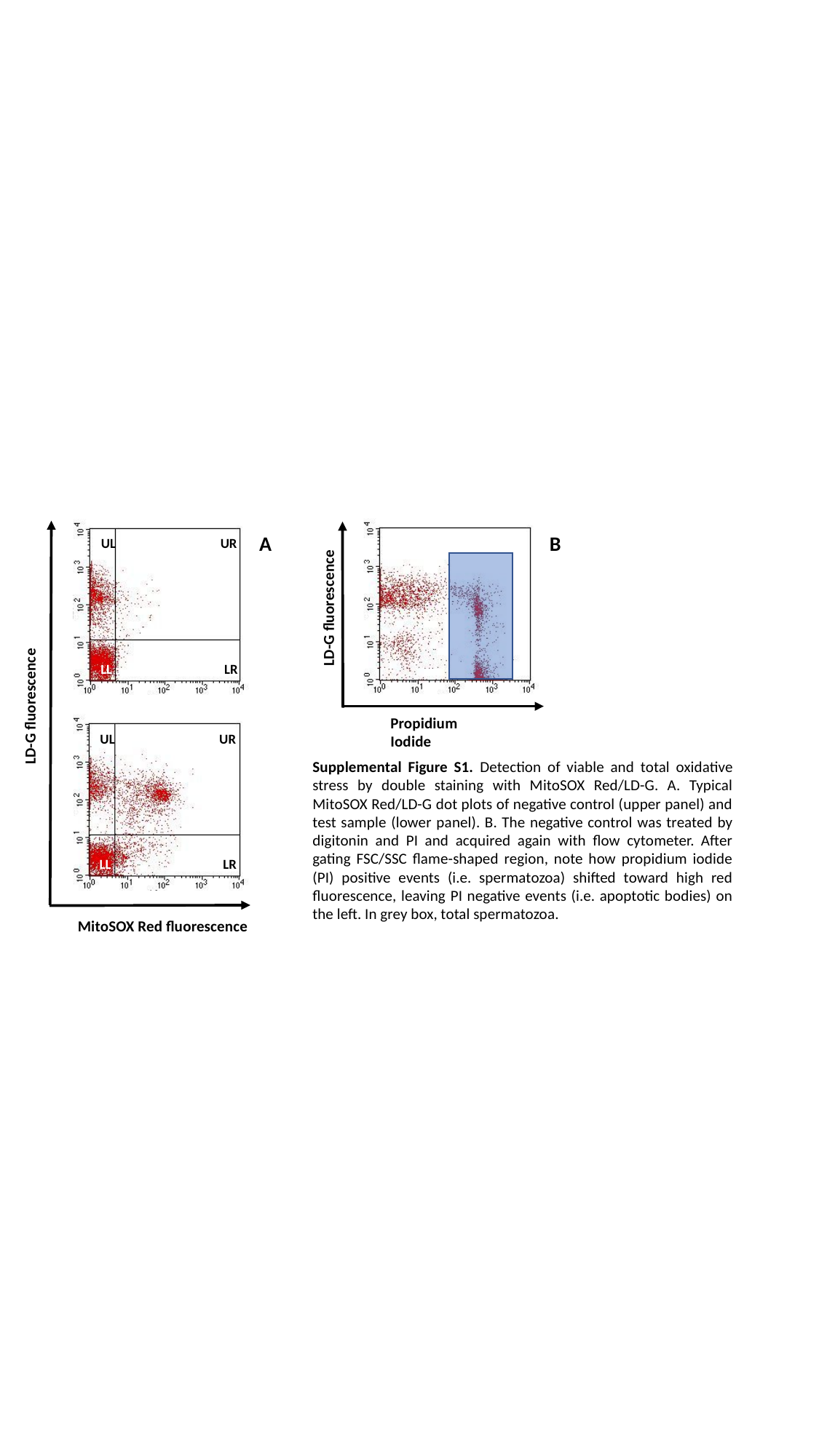

A
B
UL
UR
LD-G fluorescence
LL
LR
LD-G fluorescence
Propidium Iodide
UL
UR
Supplemental Figure S1. Detection of viable and total oxidative stress by double staining with MitoSOX Red/LD-G. A. Typical MitoSOX Red/LD-G dot plots of negative control (upper panel) and test sample (lower panel). B. The negative control was treated by digitonin and PI and acquired again with flow cytometer. After gating FSC/SSC flame-shaped region, note how propidium iodide (PI) positive events (i.e. spermatozoa) shifted toward high red fluorescence, leaving PI negative events (i.e. apoptotic bodies) on the left. In grey box, total spermatozoa.
LL
LR
MitoSOX Red fluorescence

## Slide 2
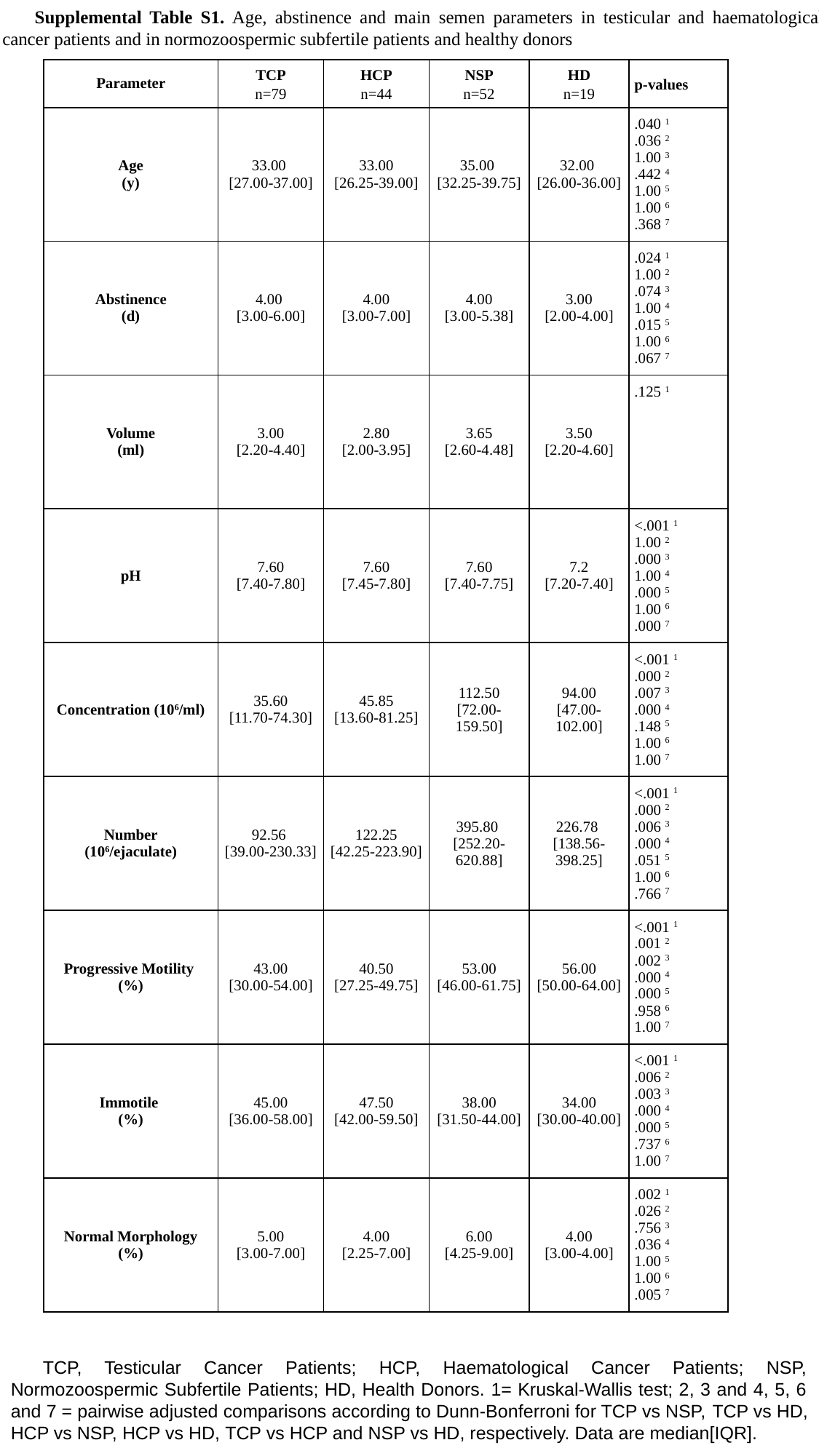

Supplemental Table S1. Age, abstinence and main semen parameters in testicular and haematological cancer patients and in normozoospermic subfertile patients and healthy donors
| Parameter | TCP n=79 | HCP n=44 | NSP n=52 | HD n=19 | p-values |
| --- | --- | --- | --- | --- | --- |
| Age (y) | 33.00 [27.00-37.00] | 33.00 [26.25-39.00] | 35.00 [32.25-39.75] | 32.00 [26.00-36.00] | .040 1 .036 2 1.00 3 .442 4 1.00 5 1.00 6 .368 7 |
| Abstinence (d) | 4.00 [3.00-6.00] | 4.00 [3.00-7.00] | 4.00 [3.00-5.38] | 3.00 [2.00-4.00] | .024 1 1.00 2 .074 3 1.00 4 .015 5 1.00 6 .067 7 |
| Volume (ml) | 3.00 [2.20-4.40] | 2.80 [2.00-3.95] | 3.65 [2.60-4.48] | 3.50 [2.20-4.60] | .125 1 |
| pH | 7.60 [7.40-7.80] | 7.60 [7.45-7.80] | 7.60 [7.40-7.75] | 7.2 [7.20-7.40] | <.001 1 1.00 2 .000 3 1.00 4 .000 5 1.00 6 .000 7 |
| Concentration (106/ml) | 35.60 [11.70-74.30] | 45.85 [13.60-81.25] | 112.50 [72.00-159.50] | 94.00 [47.00-102.00] | <.001 1 .000 2 .007 3 .000 4 .148 5 1.00 6 1.00 7 |
| Number (106/ejaculate) | 92.56 [39.00-230.33] | 122.25 [42.25-223.90] | 395.80 [252.20-620.88] | 226.78 [138.56-398.25] | <.001 1 .000 2 .006 3 .000 4 .051 5 1.00 6 .766 7 |
| Progressive Motility (%) | 43.00 [30.00-54.00] | 40.50 [27.25-49.75] | 53.00 [46.00-61.75] | 56.00 [50.00-64.00] | <.001 1 .001 2 .002 3 .000 4 .000 5 .958 6 1.00 7 |
| Immotile (%) | 45.00 [36.00-58.00] | 47.50 [42.00-59.50] | 38.00 [31.50-44.00] | 34.00 [30.00-40.00] | <.001 1 .006 2 .003 3 .000 4 .000 5 .737 6 1.00 7 |
| Normal Morphology (%) | 5.00 [3.00-7.00] | 4.00 [2.25-7.00] | 6.00 [4.25-9.00] | 4.00 [3.00-4.00] | .002 1 .026 2 .756 3 .036 4 1.00 5 1.00 6 .005 7 |
TCP, Testicular Cancer Patients; HCP, Haematological Cancer Patients; NSP, Normozoospermic Subfertile Patients; HD, Health Donors. 1= Kruskal-Wallis test; 2, 3 and 4, 5, 6 and 7 = pairwise adjusted comparisons according to Dunn-Bonferroni for TCP vs NSP, TCP vs HD, HCP vs NSP, HCP vs HD, TCP vs HCP and NSP vs HD, respectively. Data are median[IQR].

## Slide 3
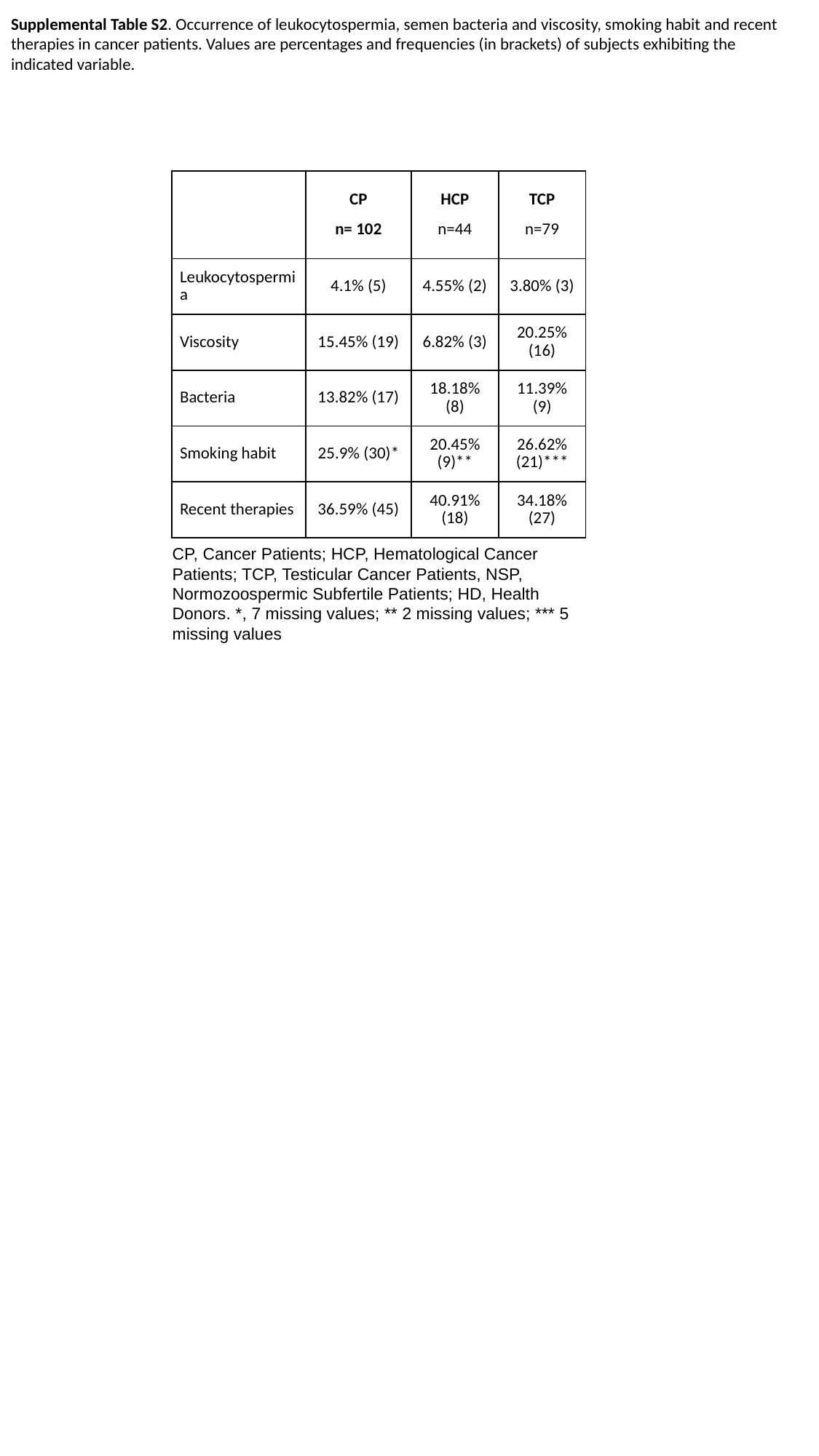

Supplemental Table S2. Occurrence of leukocytospermia, semen bacteria and viscosity, smoking habit and recent therapies in cancer patients. Values are percentages and frequencies (in brackets) of subjects exhibiting the indicated variable.
| | CP n= 102 | HCP n=44 | TCP n=79 |
| --- | --- | --- | --- |
| Leukocytospermia | 4.1% (5) | 4.55% (2) | 3.80% (3) |
| Viscosity | 15.45% (19) | 6.82% (3) | 20.25% (16) |
| Bacteria | 13.82% (17) | 18.18% (8) | 11.39% (9) |
| Smoking habit | 25.9% (30)\* | 20.45% (9)\*\* | 26.62% (21)\*\*\* |
| Recent therapies | 36.59% (45) | 40.91% (18) | 34.18% (27) |
CP, Cancer Patients; HCP, Hematological Cancer Patients; TCP, Testicular Cancer Patients, NSP, Normozoospermic Subfertile Patients; HD, Health Donors. *, 7 missing values; ** 2 missing values; *** 5 missing values

## Slide 4
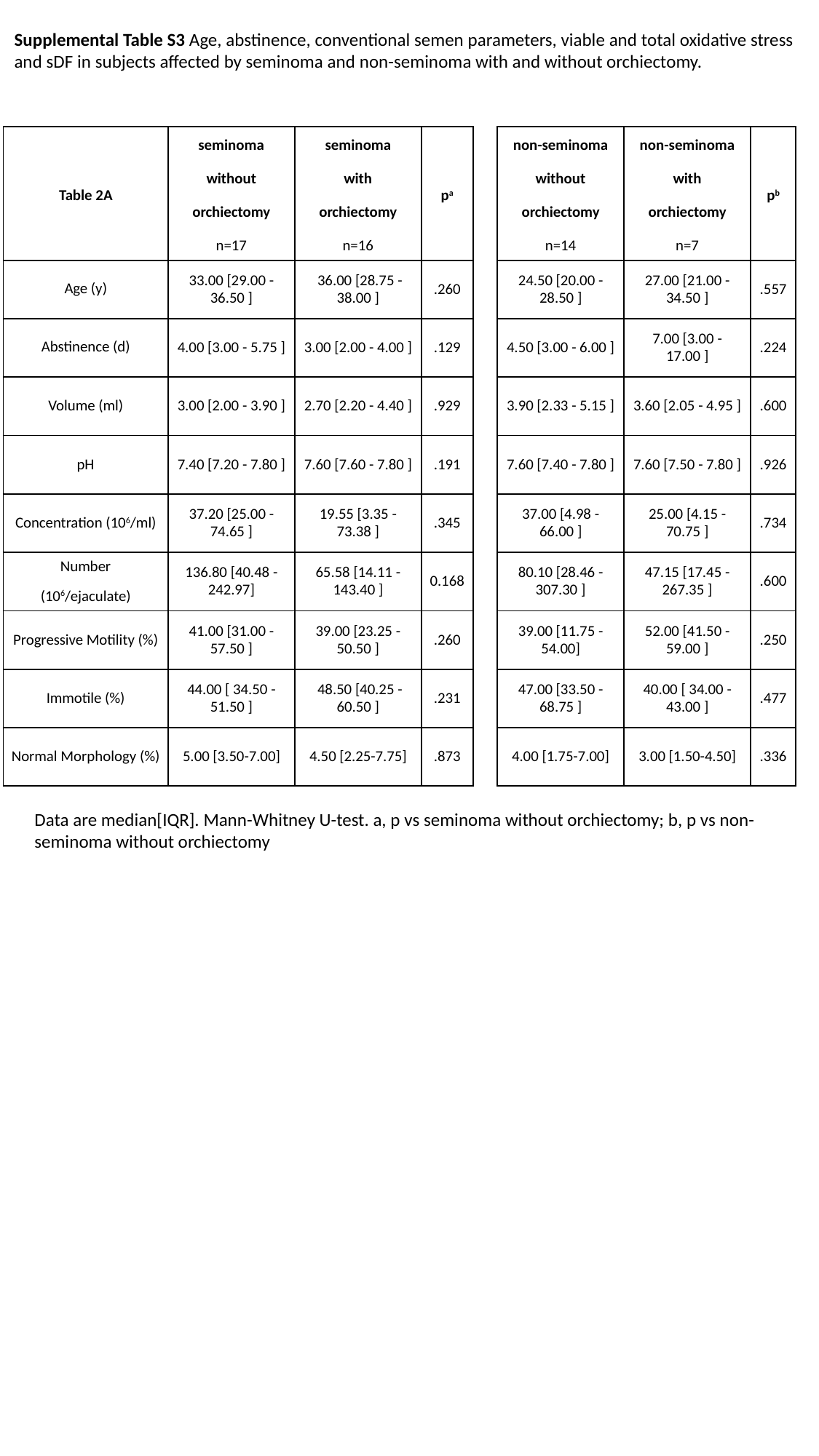

Supplemental Table S3 Age, abstinence, conventional semen parameters, viable and total oxidative stress and sDF in subjects affected by seminoma and non-seminoma with and without orchiectomy.
| Table 2A | seminoma without orchiectomy n=17 | seminoma with orchiectomy n=16 | pa | | non-seminoma without orchiectomy n=14 | non-seminoma with orchiectomy n=7 | pb |
| --- | --- | --- | --- | --- | --- | --- | --- |
| Age (y) | 33.00 [29.00 - 36.50 ] | 36.00 [28.75 - 38.00 ] | .260 | | 24.50 [20.00 - 28.50 ] | 27.00 [21.00 - 34.50 ] | .557 |
| Abstinence (d) | 4.00 [3.00 - 5.75 ] | 3.00 [2.00 - 4.00 ] | .129 | | 4.50 [3.00 - 6.00 ] | 7.00 [3.00 - 17.00 ] | .224 |
| Volume (ml) | 3.00 [2.00 - 3.90 ] | 2.70 [2.20 - 4.40 ] | .929 | | 3.90 [2.33 - 5.15 ] | 3.60 [2.05 - 4.95 ] | .600 |
| pH | 7.40 [7.20 - 7.80 ] | 7.60 [7.60 - 7.80 ] | .191 | | 7.60 [7.40 - 7.80 ] | 7.60 [7.50 - 7.80 ] | .926 |
| Concentration (106/ml) | 37.20 [25.00 - 74.65 ] | 19.55 [3.35 - 73.38 ] | .345 | | 37.00 [4.98 - 66.00 ] | 25.00 [4.15 - 70.75 ] | .734 |
| Number (106/ejaculate) | 136.80 [40.48 - 242.97] | 65.58 [14.11 - 143.40 ] | 0.168 | | 80.10 [28.46 - 307.30 ] | 47.15 [17.45 - 267.35 ] | .600 |
| Progressive Motility (%) | 41.00 [31.00 - 57.50 ] | 39.00 [23.25 - 50.50 ] | .260 | | 39.00 [11.75 - 54.00] | 52.00 [41.50 - 59.00 ] | .250 |
| Immotile (%) | 44.00 [ 34.50 - 51.50 ] | 48.50 [40.25 - 60.50 ] | .231 | | 47.00 [33.50 - 68.75 ] | 40.00 [ 34.00 - 43.00 ] | .477 |
| Normal Morphology (%) | 5.00 [3.50-7.00] | 4.50 [2.25-7.75] | .873 | | 4.00 [1.75-7.00] | 3.00 [1.50-4.50] | .336 |
Data are median[IQR]. Mann-Whitney U-test. a, p vs seminoma without orchiectomy; b, p vs non-seminoma without orchiectomy
